# PTSD Symptoms and their Relationship with Depression, Anxiety, and Physical Health in Patients undergoing Treatment for Advanced Stage Lung Cancer

**DOI:** 10.21203/rs.3.rs-5983843/v1

**Published:** 2025-02-17

**Authors:** Christopher Penn, James Gerhart, Shriya Saxena, Laurie McLouth

**Affiliations:** University of Kentucky; Ohio University; Ohio University; University of Kentucky

**Keywords:** lung cancer, post-traumatic stress disorder, anxiety, depression, pain, sleep

## Abstract

**Purpose:**

Advanced stage lung cancer (ASLC) is a leading cause of cancer mortality. Patients undergoing treatment for ASLC often experience significant comorbid psychiatric and physical distress. Given the acutely life-threatening nature of ASLC, and distressing physical symptoms, patients may be at risk for post-traumatic stress disorder (PTSD).

**Methods:**

This study investigated PTSD symptoms and their association with psychiatric comorbidities and physical health concerns among a subsample of 77 adults undergoing active treatment for ASLC as part of a cross-sectional mixed methods design.

**Results:**

Approximately 14.5 percent of the sample reported clinically significant PTSD symptoms. Symptom severity was positively correlated with anxiety and depressive symptoms (p < .001), and physical concerns including pulmonary symptoms, poor sleep quality, pain intensity, and pain-related interference.

**Conclusions:**

PTSD symptoms were elevated among patients with ASLC and uniquely linked to difficulties with sleep and pulmonary symptoms. Further assessment is needed as these symptoms are often accompanied by other psychiatric symptoms and physical health concerns that can erode well-being and quality of life.

## Introduction

In 2021, roughly 130,000 people were diagnosed with advanced stage lung cancer (ASLC) in the United States of America (CDC, 2024). Although still the leading cause of cancer mortality (Siegel et al., 2024), therapeutic advances in ASLC treatment have significantly improved survival, with roughly one-third of people with ASLC surviving five years (Schabath & Cote, 2019). Unfortunately, many people with ASLC suffer from psychological concerns that go unaddressed in cancer care (McLouth et al., 2021). Approximately 15–44% of lung cancer patients experience depression and anxiety (Yuan et al., 2024). The deleterious effects of depression are well-documented for cancer patient treatment, physical symptoms, quality of life, and even survival (Walker et al., 2021; Lei et al., 2023), yet other psychiatric concerns such as post-traumatic stress disorder (PTSD) have been comparatively understudied.

The acutely life-threatening nature of lung cancer, pain, and pulmonary symptoms combined with the tendency for individuals to engage in negative appraisal processes such as self-blame may culminate in severe stress reactions very similar to PTSD (Abbey et al., 2015; Leano et al., 2019), a trauma-related psychiatric disorder that may occur after exposure to actual or threatened death, serious injury, or sexual violence (Diagnostic and Statistical Manual of Mental Disorders: DSM-5^^™^^, 5th Ed., 2013). PTSD symptoms are grouped into four clusters including re-experiencing memories of the traumatic event (Cluster A), avoidance (Cluster B) negative alterations in cognitions and mood (e.g., Cluster C), and hyperarousal and reactivity (Cluster D). Although changes to the DSM have prompted debate about the diagnostic classification of severe medical stress and trauma, the incidence of PTSD may be higher among cancer survivors (Varela et al., 2013) as compared to the general population (Spinhoven et al., 2014; Swartzman et al., 2017). Rural individuals with lung cancer may be particularly vulnerable to PTSD symptoms if the stigmatized nature of their lung cancer is compounded by negative rural attitudes toward mental illness and challenges accessing care (Ferris-Day et al., 2021).

This study aimed to characterize PTSD symptoms and their association with depression, anxiety, and health-related quality of life in patients undergoing active treatment for advanced stage lung cancer in an academic or community setting. Secondary goals included examining whether PTSD symptoms differed by clinical (previous cancer diagnosis) and sociodemographic factors (residence, age, sex), and determining the proportion of patients with PTSD, depression, or anxiety symptoms accessing mental health care during ASLC treatment. It was hypothesized that patients undergoing treatment for ASLC would experience more severe PTSD symptoms as compared to the general population, and that these symptoms would often co-occur with symptoms of depression and anxiety. Based on prior literature, it was expected that PTSD symptoms would be more common among patients who identified as female, were younger, treated in the community (vs. academic medical center), and had a previous cancer diagnosis. Finally, it was hypothesized that individuals with more severe PTSD symptoms would report greater difficulties with sleep quality, pulmonary symptoms, pain intensity, and pain-related activity interference.

## Method

### Participants

Participants were recruited as part of a larger study aimed at understanding attitudes and experiences with palliative and supportive care services among patients with ASLC who reside primarily in rural communities. Of 110 participants recruited for the study, 77 participants successfully completed the surveys, with a survey completion rate of 70% (77/110) (McLouth et al., 2023). Sample demographics and reasons for refusal have been previously reported (McLouth et al., 2024). The sample consisted of 77 adults (Mean age = 64.69 years old; SD = 10.31; r = 39–88). Patients were predominantly White (96%), from rural areas (62%), and the highest education attained was a high school degree (59%) (Table 1).

This study was approved by the University of Kentucky Medical Institutional Review Board (#55171) and conducted in accordance with the Declaration of Helsinki. Study inclusion criteria was based on age and treatment status. Patients over 18 who were undergoing treatment for ASLC were included. Study exclusion criteria were the inability to speak English or any documented cognitive impairment or psychiatric concern. Data were collected as part of a comprehensive analysis of current practices, barriers, and facilitators in palliative care during treatment for ASLC. Eligible patients were approached in the clinic or via the telephone and the study was described. Informed consent was collected verbally from interested patients. Screening measures for depression, PTSD, and anxiety were administered in-person or through REDCap, an online survey platform. Participants were compensated $30 for study participation.

### Measures

#### Demographics.

Self-reported demographic components included patient age, sex, race, ethnicity, and educational status. Information regarding current and prior lung cancer treatments was also selfreported. Patients’ rural status was identified using ZIP codes and the Federal Office of Rural Health Policy’s database of eligible ZIP codes (https://www.hrsa.gov/rural-health/about-us/definition/datafiles.html). Data regarding the stage of lung cancer was extracted from the electronic health record.

#### Post-Traumatic Stress Symptom Checklist.

The PTSD Checklist for the DSM-5 (PCL-5) (Blevins et al., 2015) was employed to assess severity of PTSD symptoms and provide a provisional PTSD diagnosis. The PCL-5 is a 20-item self-report measure with a rating scale of 0 indicating “Not at all” to 4 indicating “Extremely.” A summation of scores range from 0 to 80, with higher scores indicating greater PTSD symptom severity (Blevins et al., 2015). Studies indicate that a total score of 33 or higher reflects a probable PTSD diagnosis (Blevins et al., 2015). PCL-5 scores have been reported as having high internal consistency (α = .94), good test-retest reliability (r = .82), and convergent and discriminant validity (Blevins et al., 2015). Scores were internally consistent in the current sample (α = .96; subscale α = .76 to .82). Analyses based on criteria for each symptom cluster of PTSD were also conducted. Patients were assessed on criteria A, B, C and D as prescribed by the DSM-5 for PTSD. Lastly, we examined the proportion of patients reporting individual PTSD symptoms at a “2” or higher (i.e., moderately, or above).

#### Patient Reported Measurement Information System.

PROMIS (Hays et al., 2018) assessed health-related quality of life. PROMIS short-forms assessed anxiety, depression, sleep quality, and pain-related activity interference. Participant ratings were on a scale of 1 (e.g., never; not at all) to 5 (e.g., always; very much). Pain intensity was assessed with a single item rated on a scale of 0 (no pain at all) to 10 (worst pain imaginable). Probable anxiety and depression were identified by T-scores of 60 or higher. The short-form scores were internally consistent in the current sample (α = .84 to .94).

#### Functional Assessment of Cancer Therapy - Pulmonary Symptom Index.

**FACT-PSI** (Cella et al., 2011) assessed pulmonary symptoms such as shortness of breath. Participants used a scale of 1 (not at all) to 5 (very much) to rate the following statements: “I have been short of breath,” “I have been coughing,” “I feel tightness in my chest,” and “Breathing is easy for me” (reverse-scored). FACT-PSI scores were internally consistent in the current sample (α = .81).

#### Mental Healthcare Utilization.

This measure was assessed via self-report. Participants indicated whether they had received services from a psychologist, psychiatrist, or social worker while receiving treatment for ASLC.

## Analysis

Data were analyzed using SPSS version 29 (IBM SPSS Statistics for Windows, 2023). Descriptive statistics were computed to characterize the sociodemographic characteristics of the sample. Differences in PTSD scores across demographic groups were assessed using independent samples t-tests. Association of probable PTSD diagnostic status with other psychiatric history was assessed with chi-square tests. Correlations and multiple regression were used to assess associations between health-related quality of life and PTSD severity.

## Results

### PTSD Symptoms and Probable PTSD

The mean PCL score of this sample was 15.42 (SD = 15.84, Median = 11.5, IQR 4 to 22.5) with scores ranging from 0 (n = 7) to 80 (n = 1). The most reported PTSD symptom was “Trouble falling or staying asleep” (40.8%; arousal and reactivity symptom), followed by “Feeling distant or cut off from other people” (39%; negative alterations in cognition and mood symptom), and “Loss of interest in activities that you used to enjoy” (35.5%; negative alterations in cognition and mood symptom). The least reported PTSD symptom was “Taking too many risks or doing things that could cause you harm” (6.6%; arousal and reactivity symptom) followed by “Blaming yourself or someone else for the stressful experience or what happened after it” (11.7%; negative alterations in cognition and mood symptom).

Using a cutoff score of 33 or higher, 14.5% (n = 11) met criteria for probable PTSD; 36.4% (n = 28) met DSM-5 criteria for cluster D symptoms (i.e., negative alterations in cognition and mood); 35.5% (n = 27) met criteria for cluster B (i.e., intrusion symptoms); 32.5% (n = 25) met criteria for cluster E (i.e., arousal and reactivity); and 27.6% (n = 21) met criteria for cluster C (i.e., avoidance).

## Clinical and Sociodemographic Subgroups

Mean scores on the PCL did not differ significantly across patient sex (Mean = 14.2 females vs. 16.6 males, p = 0.492), residence (Mean = 15.5 rural vs. 15.4 urban, p = .477), treatment site (Mean = 15.9 community vs. 14.7 academic, p = 0.713), or previous cancers (Mean = 14.9 multiple cancers vs. 15.8 one cancer, p = .844). Likewise, the proportion of patients who met criteria for probable PTSD was not significantly different by sex (females = 10.8% vs. males = 17.9%, p =0.518), residence (rural = 12.5% vs. urban = 17.9%, p = 0.521), treatment site (community = 15.9% vs. academic = 12.5%; p =0.752), or previous cancers (multiple cancers = 10.7% vs. one cancer = 16.7%; p = 0.737).

### Depression, Anxiety, and Correlations with PTSD Symptoms and Probable PTSD

Within our sample, 37.7% (n = 29) met criteria for clinically significant depression or anxiety. Approximately one-quarter of the patients screened positive for depression (26%, n = 20) and over 30% screened positive for anxiety (31.2%, n = 24). Anxiety and depression severity scores were strongly associated with total PTSD severity and cluster severity scores (rs = .62 to .76, p < .001). Patients who screened positive for depression and anxiety had significantly higher mean PCL scores than those without depression (Mean PCL scores for patients with clinically significant depression = 31.8 vs. without = 10.0, p < .001) and anxiety (Mean PCL scores for patients with clinically significant anxiety = 30.0 vs. without = 9.1, p< .001). Of the 29 patients who screened positive for clinically significant depression or anxiety, 32.1% (n = 9) also met criteria for probable PTSD. Of the 26 patients with depression, 42.1% (n = 8), p< .001 screened positive for probable PTSD. Of the 24 patients with anxiety, 39.1% (n = 9), p< .001 also met criteria for probable PTSD.

## Psychopathology by Service Utilization

Within our total sample, 40.8% (n = 31) of patients met the criteria for probable PTSD, depression, or anxiety. Of this subsample, 12.9% (n = 4) had seen a psychiatrist, 16.1% (n = 5) had seen a psychologist, and 48.4% (n = 15) had seen a social worker since starting ASLC treatment. Only one patient indicated seeing a psychiatrist on a regular basis, and one other patient indicated seeing a psychologist on a regular basis.

## Associations with Health-Related Quality of Life

As hypothesized, poorer sleep quality (r = − .57, p < .001), greater pain intensity (r = .28, p < .018), pain-related activity interference (r = .34, p < .001), and pulmonary symptoms (r = .51, p< .001). were significantly associated with PCL scores.

The final analysis assessed these health-related quality of life concerns as unique predictors of PCL scores. Items assessing nightmares and difficulty sleeping were removed from the PCL score to avoid overlap of poor sleep quality with sleep-specific PTSD symptoms. In a linear regression accounting for 34.9% of the variance in PCL scores, (F = 4.69 = 9.24, p < .001), pulmonary symptoms (β = .25, p = .021), and poor sleep quality (β = .42, p < .001) were significantly associated with PCL scores (Table 2). In contrast, pain intensity (β = .02, p = .912) and pain interference (β = .06, p = .701) were no longer associated with PCL scores. Thus, a greater burden of physical health concerns, particularly difficulties with breathing and sleep, were linked to higher PCL scores.

## Discussion

Advanced stage lung cancer patients may be at increased risk for PTSD symptoms due to the severity of the illness compounded by its disproportionate incidence among potentially under-resourced populations. The study aimed to examine the prevalence of PTSD symptoms and their co-occurrence with depression and anxiety among patients receiving treatment for ASLC in both an academic and a community cancer care setting. Using a cutoff score of 33 or greater, 14.5% (n = 11) of our sample met the criteria for probable PTSD. This observed rate is higher than the estimated 6–8% lifetime prevalence of the general population and comparable to the prevalence rates of PTSD in adult cancer survivors and individuals employed in high stress occupations (Abbey et al., 2015; Kangas et al., 2002; Morrison et al., 2021; Smith et al., 2024; Swartzman et al., 2017).

Although PTSD is a heterogeneous disorder (Galatzer-Levy & Bryant, 2013) and highly comorbid with emotional disorders, one-fifth to one-quarter of the sample noted uniquely trauma-specific symptoms including repeated, disturbing, and unwanted memories (26%), avoidance of these memories, thoughts, or feelings (24%), and the external reminders of the stressful experience (20%) not captured by anxiety and depression diagnoses. Probable psychiatric comorbidities of anxiety and depression were common, with over 35% of the sample experiencing clinically significant levels of anxiety and/or depression.

Very few patients who screened positive for probable PTSD, anxiety, or depression reported receiving services from a mental health provider during their ASLC treatment. Given the previously low rates of supportive care service utilization among patients with ASLC (Mosher et al., 2013), it was unclear if social work services reported in the sample targeted case management (e.g., at intake for social needs assessment, financial counseling) or psychosocial distress. Only two patients with probable diagnoses reported seeing a psychiatrist or psychologist on a regular basis.

Contrary to expectations, sociodemographic and clinical factors were not associated with PTSD symptoms or probable PTSD in this sample. No differences were found in the proportion of patients with probable PTSD or PTSD symptom severity based on sex, rural/urban residential status, location of treatment facility, or lifetime number of cancers. The study did, however, replicate and extend prior work on the physical symptom correlates of PTSD symptoms in cancer populations (Lillis et al., 2018; Swartzman et al., 2017; Varela et al., 2013). Pulmonary symptoms, poor sleep quality, pain intensity, and pain-related activity interference were all associated with PTSD severity. Regression analysis indicated that pulmonary symptoms and poor sleep quality were unique predictors. Taken together, physical status and quality of life were more relevant to understanding PTSD symptoms than static background factors.

The findings of this study should be interpreted within the context of the study’s strengths and limitations. Data were gathered from a targeted sample of community dwelling adults with ASLC using previously validated measures. As the study was conducted with a small, targeted sample, statistical power was limited. The cross-sectional design precludes causal inference, and further information is needed about the time course of potential prior trauma exposure and psychiatric history. Thus, future investigations should employ prospective methods to assess how PTSD symptoms develop in response to various phases of cancer care (e.g., diagnosis, treatment, remission) to better understand the perceived trauma/stressor and related course of symptoms.

## Conclusion

Expanding psychological assessments to incorporate screening for PTSD symptoms may improve healthcare providers’ abilities to identify psychopathology which, if left unaddressed, could have deleterious effects on patients’ quality of life and cancer care. Together, these results underscore the importance of expanding screening to assess psychopathology beyond depression and anxiety. Therefore, it may be useful to administer additional assessments to patients who screen positive for distress. Brief measures such as the four-item version of the PCL-5 or the Primary Care PTSD Screen (PC-PTSD-5) may be used in oncology settings to identify possible cases for further evaluation with structured assessments by mental health professionals (Smith et al., 2024; Supporting Cancer Patients with Post-Traumatic Stress Disorder (PTSD) / NCCN Continuing Education, n.d.). Interdisciplinary treatments that leverage and synergize resources from palliative and supportive care specialists with community-based mental health providers may be needed to expand services to this and other at-risk groups (Strada, 2018).

## Figures and Tables

**Table 1:** Sociodemographic and Clinical Characteristics of Patients (N = 77)

Demographics	n	%
Race / Ethnicity		
White / Caucasian	74	96.1
Black / African American	2	2.6
Missing	1	1.3
Female	38	49.4
Married / Partnered	53	68.8
Rural^a^	48	62.3
Highest Education Level		
< Grade 12 completed	16	20.8
Graduated high school or equivalent	29	37.7
Post-high school training	8	10.4
Some college	11	14.3
College graduate or more	12	15.6
Missing	1	1.3

a Per Federal Office of Rural Health Policy.

Note: Demographic and clinical variables previously reported in McLouth et al., 2023. Palliative care use and utilization determinants among patients treated for advanced stage lung cancer care in the community and academic medical setting. Support Care Cancer. 2023 Feb 27;31(3):190. doi: 10.1007/s00520-023-07649-y. PMID: 36847880; PMCID: PMC9969037

**Table 2:** Health-Related Quality of Life and PTSD Symptom Severity

	B	SE	β	t	p
Pulmonary Symptoms	4.65	1.96	.25	2.37	.021
Sleep Quality	1.47	.39	.42	3.81	< .001
Pain Interference	.60	1.56	.06	.39	.701
Pain Intensity	.08	.71	.02	.11	.912

Note: PCL Score computed without sleep and nightmare items.

## Data Availability

Data will be made available upon reasonable request to the corresponding author.
